# Hypoxia-induced MTA1 promotes MC3T3 osteoblast growth but suppresses MC3T3 osteoblast differentiation

**DOI:** 10.1186/s40001-015-0084-x

**Published:** 2015-02-03

**Authors:** Tielong Liu, Weiwei Zou, Guodong Shi, Jian Xu, Fei Zhang, Jianru Xiao, Yan Wang

**Affiliations:** Department of Orthopaedics, Shanghai Changzheng Hospital, 415, Fengyang Road, Shanghai, 200003 China; Department of Medical Imaging, Shanghai Changzheng Hospital, Shanghai, 200003 China; Suqian Worker’s Hospital, Suqian, Jiangsu Province 223800, China; Center Hospital of Ningbo Development Zone, Ningbo, Zhejiang Province 315800, China; Department of Orthopaedics, The General Hospital of People’s Liberation Army, 28, Fuxing Road, Haidian District, Beijing, 100853 China

**Keywords:** Bone fracture, Fracture healing, Hypoxia-induced MTA1, Osteoblast cell growth, Osteoblast differentiation

## Abstract

**Background:**

Bone fracture is one of the most common physical injuries in which gene expression and the microenvironment are reprogramed to facilitate the recovery process.

**Methods:**

By specific siRNA transfection and MTT assay, we evaluated the effects of metastasis-associated gene 1 (MTA1) in osteoblast growth. To show the role of MTA1 in osteoblast under hypoxia conditions, by overexpressing and silencing MTA1 expression, we performed mineral deposition and alkaline phosphatase activity assay to observe the differentiation status of osteoblast cells. Real-time PCR and Western blot assays were adopted to detect the expression of certain target genes.

**Results:**

Here, we reported that hypoxia-induced MTA1 expression through hypoxia-induced factor 1 alpha (HIF-1α) and stimulated the growth of osteoblast MC3T3 cells. Silencing of MTA1 through specific siRNA suppressed MC3T3 cell growth and elicited cell differentiation and induced alkaline phosphatase activation and the upregulation of bone morphogenetic protein-2 and osteocalcin.

**Conclusions:**

We found that MTA1 was regulated by HIF-1α in hypoxia circumstance to suppress osteoblast differentiation. These findings provide new insights for bone fracture healing and new strategies to develop potential targets to promote fracture healing.

**Electronic supplementary material:**

The online version of this article (doi:10.1186/s40001-015-0084-x) contains supplementary material, which is available to authorized users.

## Background

Bone fracture is a prevalent medical condition that occurs in a wide age range of individuals. Bone fracture healing requires a certain period of time to rebuild its structure and function. Fracture causes an urgent alteration of the bone microenvironment, with a decrease in blood supply and subsequent hypoxia. The healing process of fractures starts when a hematoma forms surrounding the injured bone. However, the healing process of fracture is promoted or hindered by several factors which mainly affect the growth and differentiation of osteoblast and the mineralization of the collagen matrix. Hypoxia is one of the most prominent outcomes following fractures, significantly influencing their healing process. A recent study has demonstrated that fracture healing is delayed under hypoxic conditions [1]. Hypoxia stimulates the expression of a variety of genes, including transcriptional factors and cytokines [2]. Hypoxia-inducible factor (HIF) family members are key molecules in the hypoxia-induced network. Hypoxia induces vascular endothelial growth factor (VEGF) gene transcription in human osteoblast-like cells through HIF-2α [3]. Hypoxia induced by fracture significantly upregulates the expression of bone morphogenetic protein 2 (BMP-2), an osteogenic cytokine at the fracture site, in the capillary endothelial cells [4,5]. It is also suggested that endothelial cells may play a role in stimulating MC3T3 cells through the potent osteogenic factor BMP-2, other than in promoting angiogenesis.

Transcriptional responses to hypoxia are primarily mediated by HIF-1, a heterodimer of HIF-1, and the arylhydrocarbon receptor nuclear translocator subunit. Loss of HIF-1 eliminates almost all oxygen-regulated transcriptional responses [6]. Low oxygen tension-induced HIF-1 expression promotes osteogenic growth and differentiation, though its mechanism is not fully understood.

Metastasis tumor antigen 1 (MTA1) is a component of the chromatin remodeling complex which influences gene transcription by modulating the target gene chromatin [7]. MTA1 has been reported to be upregulated in a series of carcinomas [8]. Overexpression of MTA1 in cancer cells enhances cancer cell growth. Additionally, MTA1 is also involved in the function of DNA damage checkpoints [9] and anoikis [10]. It has been reported that MTA1 stabilizes HIF-1 by recruiting histone deacetylase-1 [11]. However, whether MTA1 was regulated by HIF-1 has never been illustrated. Moreover, the biological function of MTA1 in osteoblast cells, especially in bone fracture microenvironment, has not been reported. How MTA1 and HIF-1 interact with each other in the context of fracture-associated hypoxia circumstances is to be demonstrated.

In the present study, we analyzed the transcriptional profiling after hypoxia stress in MC3T3 cells, and found MTA1 was significantly upregulated. We further investigated the possible role of MTA1 in the fracture healing process and its mechanism.

## Methods

### Cell lines and culture conditions

The MC3T3 cell line was obtained from the American Type Culture Collection (Manassas, VA, USA) and was routinely maintained in α-modified Eagle’s Minimum Essential Medium complete medium supplemented with 10% fetal bovine serum (Gibco, Life Technologies, Grand Island, NY, USA) before exposure to hypoxia. For hypoxia treatment, cells were cultured at 37°C under 1% O_2_ tension, with 95% N_2_ and 5% CO_2_ (Anaerobic System PROOX model 110; BioSpherix Ltd., Lacona, NY, USA).

### MTA1 plasmid and siRNA transfection

The MTA1 coding sequence was inserted into pCDH-CMV-MCS-EF1-copGFP vector (pCDH-MTA1) by using endonucleotases EcoRI and BamHI. The cloning primers for MTA1 cDNA were as follows: forward primer: 5′-AAAGAATTCATGGCCGCCAACATGTACA-3′; reverse primer: 5′-AAAGGATCCCTAGTCCTCAATAACAATGGGCTC-3′. Small interfering RNA targeting MTA1 was designed and synthesized by Shanghai Gene Pharma (Shanghai, China). The MTA1 siRNA sequences were 5′-CTTGTGCCGTGAGATCCTAdTdT-3′ and 5′-GAACACGGCACTCTAGGATdTdT-3′. The control siRNA sequences were 5′-GACTTCATAAGGCGCATdTdT-3′ and 5′-ATGCGCCTTATGAAGTCdTdT-3′. The MTA1 vector and siRNA were transfected with lipofectamine 2000 (11668-019, Invitrogen, USA) as instructed by the manual. Briefly, one day before transfection, the cells were reseeded at 30% confluence; 24 hours later, for vector DNA transfection, the complex was prepared using a DNA (μg) to lipofectamine 2,000 (μL) ratio of 4 μg/6 μL, while for siRNA transfection, the lipofectamine 2,000 and siRNA complex was prepared using a RNA (μg) to lipofectamine 2,000 (μL) ratio of 100 μg/8 μL. The complexes were mixed into 0.5 mL serum-free culture medium for 15 minutes before being added into the cells.

### Real time PCR

Total RNA isolated from treatment and ventricles of MC3T3 cells were analyzed by real-time RT-PCR. All samples were amplified in triplicates. One microgram of RNA was used to reversely transcribe cDNA (Clontech, Palo Alto, CA, USA). Relative quantification was achieved by the comparative 2^-∆∆Ct^ method. PCR reactions were performed on an ABI PRISM 7900 HT Sequence Detection system with FastStart Universal SYBR Green Master (Rox; Roche, Indianapolis, IN, USA) according to the manufacturer’s instructions. The PCR conditions were 95°C for 10 min followed by 40 cycles of 95°C for 30 sec and 60°C for 60 sec. All samples were run in triplicate and normalized to internal control GAPDH (NM_001289726).

### Western blot

Whole-cell protein extracts were prepared and quantified by the Bicinchoninic Acid Protein Assay Kit (Pierce, Rockford, IL, USA). Briefly, 100 μg of proteins were denatured, separated on 12% SDS PolyAcrylamide Gel Electrophoresis gels, and transferred onto nitrocellulose membranes (Bio-Rad, Waltham, MA, USA). The membranes were probed with primary antibodies overnight at 4°C and incubated with secondary horseradish peroxidase-conjugated secondary antibodies for 1 h at room temperature. The proteins were detected by enhanced chemoluminescence (Applygen Technologies Inc., Beijing, China) and exposed by autoradiography. To control protein loading, membranes were stripped and reprobed with GAPDH antibody (1:5,000). The primary antibodies included anti-osteocalcin (1:1,500, AB10911, Millipore, Billerica, MA, USA), anti-BMP-2 antibody (1:2,000, 18933-1-AP, Proteintech, Chicago, IL, USA), anti-alkaline phosphatase antibody (1:3,000, ab95462, Abcam, Cambridge, MA, USA), anti-MTA1 antibody (1:1,000, ab71153, Abcam), and anti-GAPDH antibody (G9545, Sigma, St. Louis, MO, USA). The expression of proteins was quantified with Image J software using actin as the internal control.

### MTT assay

The adherent cells were transfected with MTA1-specific siRNA, control siRNA, or MTA1-overexpressing vector, and were then digested and prepared into suspension before they were seeded in a 96-well plate and incubated for 4 days. The cell growth was evaluated every day. Briefly, 100 μL of MTT (0.5 mg/mL final concentration) was added and incubated in dark for an additional 4 h to induce the production of formazan crystals at 37°C. The supernatant was discarded. The formazan crystal was dissolved in dimethyl sulfoxide. Cell viability was examined on a Microplate reader (Bio-rad, Model 680) at 570 nm excitatory emission wavelength. The cell viability was plotted according to optical density values.

### Mineral deposition

Overall, 5 × 10^3^ MC3T3 cells were seeded into 6-well plates coated with 0.1% gelatin and cultured in differentiation medium consisting of α-minimal essential medium (Invitrogen, USA) supplemented with 10% (v/v) fetal bovine serum (Gibco), penicillin-streptomycin solution (with final concentration of 500 unit/mL penicillin G sodium and 500 mg/mL streptomycin sulfate; Invitrogen), 50 mg/mL ascorbic acid (Sigma), vitamin C 50 μg/mL, 10 mM b-glycerophosphate (Sigma), and 10 μmol/L dexamethasone (Sigma). The media was changed every 3 days. Mineral deposition was assessed by staining with alizarin-red. Briefly, cells were washed once in PBS and fixed in phosphate-buffered formalin for 20 min. Fixed cells were washed in PBS and subsequently stained in 1% alizarin-red S (Sigma) in distilled water for 15 min, then washed to remove the remaining dye and air-dried. Images of stained cells were captured using a light microscope (Leica, Solms, Germany) and analyzed by appropriate image software. Stained plates were photographed using a digital camera. The numbers of bone nodules were counted accordingly. Mineral deposition was also assessed by staining in alizarin-red after 14 days.

### Alkaline phosphatase (ALP) activity assay

The ALP activity was assayed with Alkaline Phosphatase Assay Kit (Colorimetric) from Abcam (ab83369). The assay was performed following the manual instructions. Briefly, 1 × 10^5^ cells were homogenized in the assay buffer, centrifuged at 13,000 *g* for 3 min to remove insoluble material, and 80 μL of each sample were added into 96-well plate and mixed with 50 μL 5 mM *p*NPP solution. The plate was incubated for 60 minutes at 25°C in the dark, until 20 μL stop solution was added into each well, except to the background control. The standard curve was also performed in this assay. The optical density value was measured at 405 nm in a microplate reader.

### Statistics

The differences in the results between groups were compared using ANOVA or Student’s *t*-test. Results are expressed as the means ± SD. SPSS 18.0 Statistical program (IBM SPSS, USA) was used for the statistical analysis. A *P* value <0.05 was considered significant.

## Results

### Hypoxia up-regulates MTA1 expression

**Figure 1:**
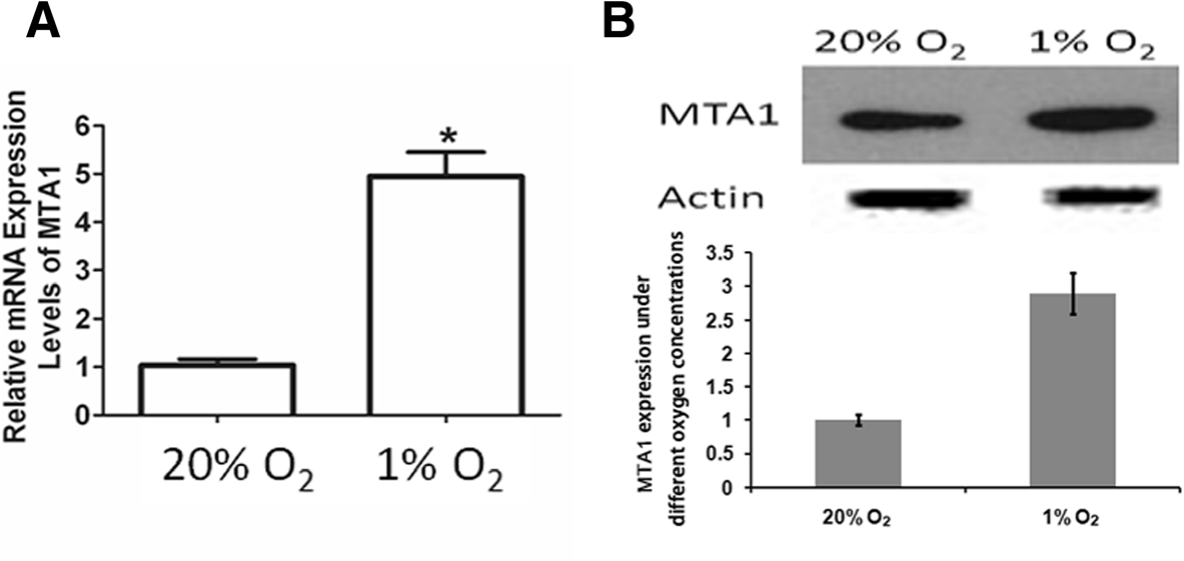
**Hypoxia upregulated MTA1 expression.** The MC3T3 cells were cultured under 1% oxygen tension (vs. 20% oxygen in the control). The expressions of MTA1 mRNA **(A)** and protein **(B)** were evaluated by real-time and Western blot, respectively (n = 3).

To examine the changes of gene profiling to hypoxia, MC3T3 cells were treated with 1% oxygen (versus 20% of control group), and the transcriptome analysis was performed by microarray (Additional file 1). We observed that MTA1 was significantly upregulated in the low-oxygen treated samples (data not shown). To verify the upregulation of MTA1 by hypoxia in MC3T3 cells, we performed real-time PCR and Western blot to check the MTA1 expression at mRNA and protein levels. Results showed that hypoxia at 1% oxygen tension significantly upregulated MTA1 expression at both mRNA and protein levels (Figure 1).

### Hypoxia upregulated MTA1 expression through HIF-1α

**Figure 2:**
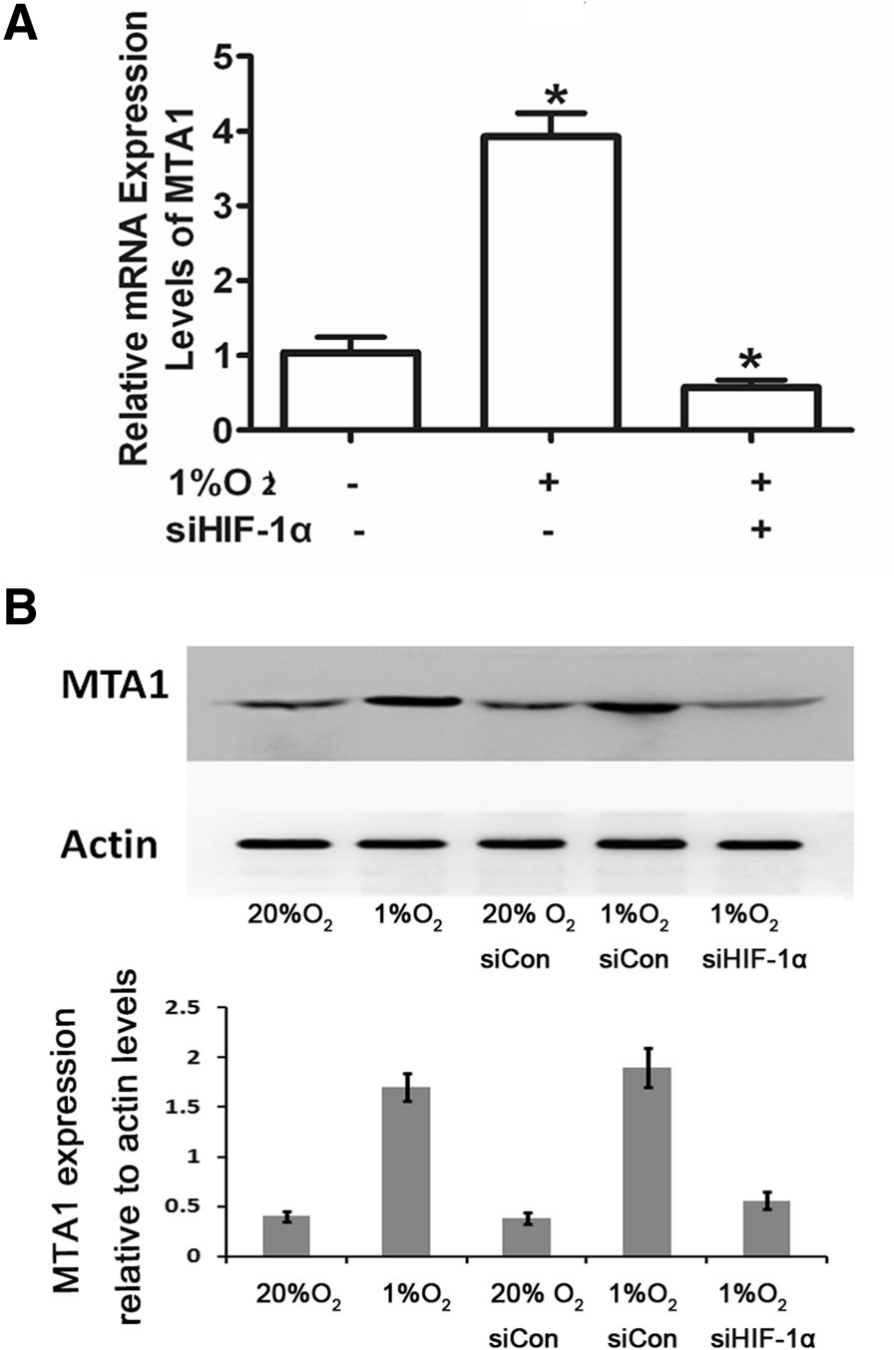
**Hypoxia upregulated MTA1 expression through HIF-1. (A)** Silencing of HIF-1α reversed the hypoxia-induced MTA1 upregulation at mRNA level (*P* <0.05). **(B)** Silencing of HIF-1 reversed the hypoxia-induced MTA1 upregulation at protein level (n = 3).

Hypoxia is a stress that can activate many signaling pathways. HIF-1 is one of the key intermediate effectors of hypoxia and translationally regulates the expression of a series of downstream genes. To explore the possible intermediate factor HIF-1 for regulating MTA1 response to hypoxia, we transfected the MC3T3 cells with HIF-1 siRNA along with hypoxia treatment, we found that the upregulation of MTA1 mRNA and protein was recovered to the level comparable to control sample (Figure 2), suggesting that HIF-1 is an activator of MTA1 expression under hypoxia.

### Hypoxia-induced MTA1 expression stimulates the growth of osteoblasts

**Figure 3:**
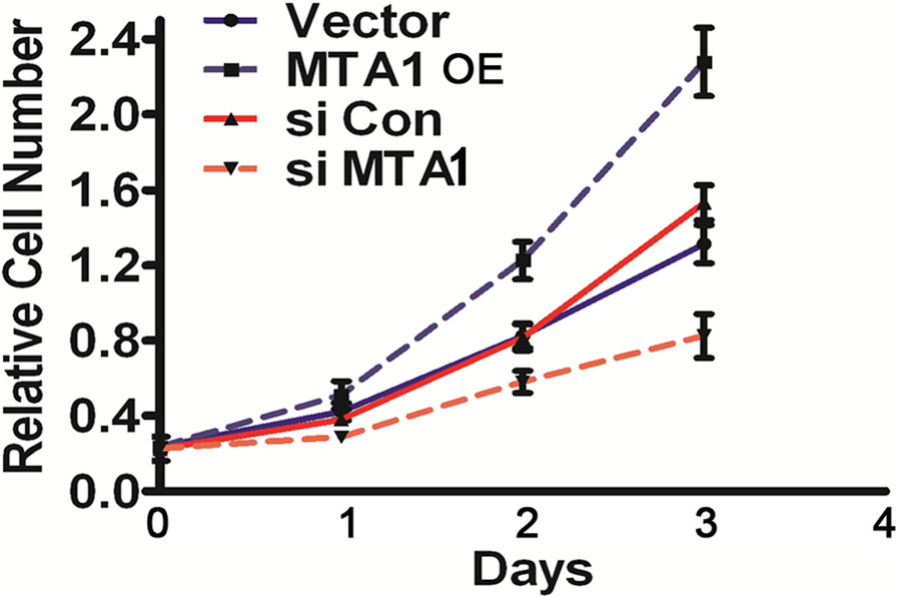
**Hypoxia-induced MTA1 promoted the growth of MC3T3 cells.** MC3T3 cells were transfected with pCDH-MTA1, MTA1 siRNA, or control siRNA. The growth of MC3T3 cells was evaluated by MTT assay. MTA1 OE, MTA1 overexpression. *P* <0.05 was considered statistically significant (n = 3).

To investigate the physiological role of MTA1 on the proliferation of osteoblasts, we performed the growth assay of MC3T3 cells under hypoxia combined with MTA1 silencing or overexpression. MTA1 overexpression significantly promoted MC3T3 cell growth (*P* <0.05), while silencing of MTA1 inhibited the growth of MC3T3 osteoblast cells (*P* <0.05). Control siRNA did not alter the growth of MC3T3 cells (*P* >0.05; Figure 3). These results indicate that, upon hypoxia, MTA1 promotes the growth of osteoblast cells.

### Silencing of MTA1 stimulates osteoblast differentiation

**Figure 4:**
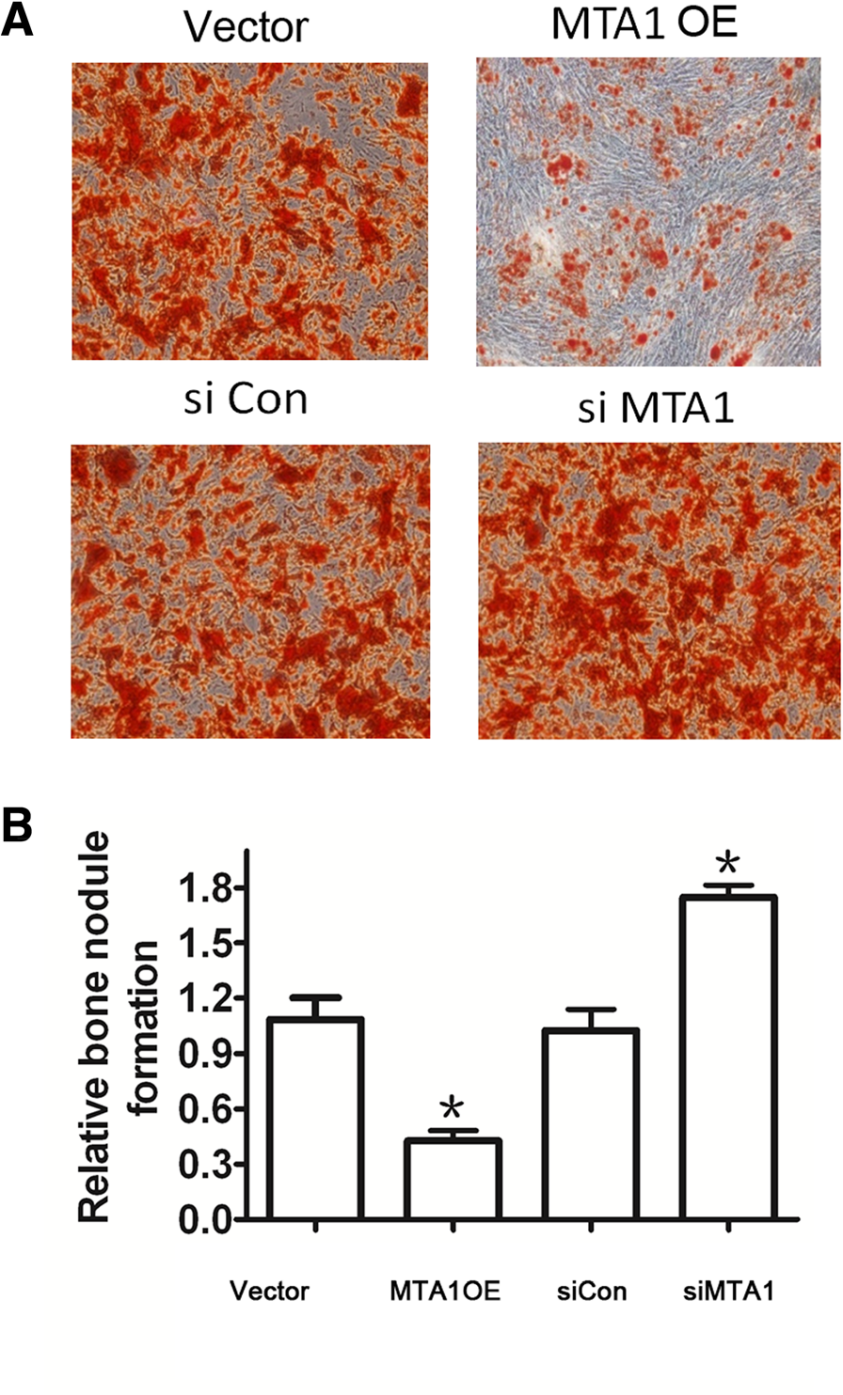
**Silencing of MTA1 slowed the mineralization of osteoblasts. (A)** Phase-contrast microscopic images of MC3T3 cells cultured in differentiation medium after overexpression or silencing of MTA1 (×100). MTA1 OE, MTA1 overexpression. **(B)** The quantitative analysis of bone nodules after MTA1 manipulation (n = 3).

**Figure 5:**
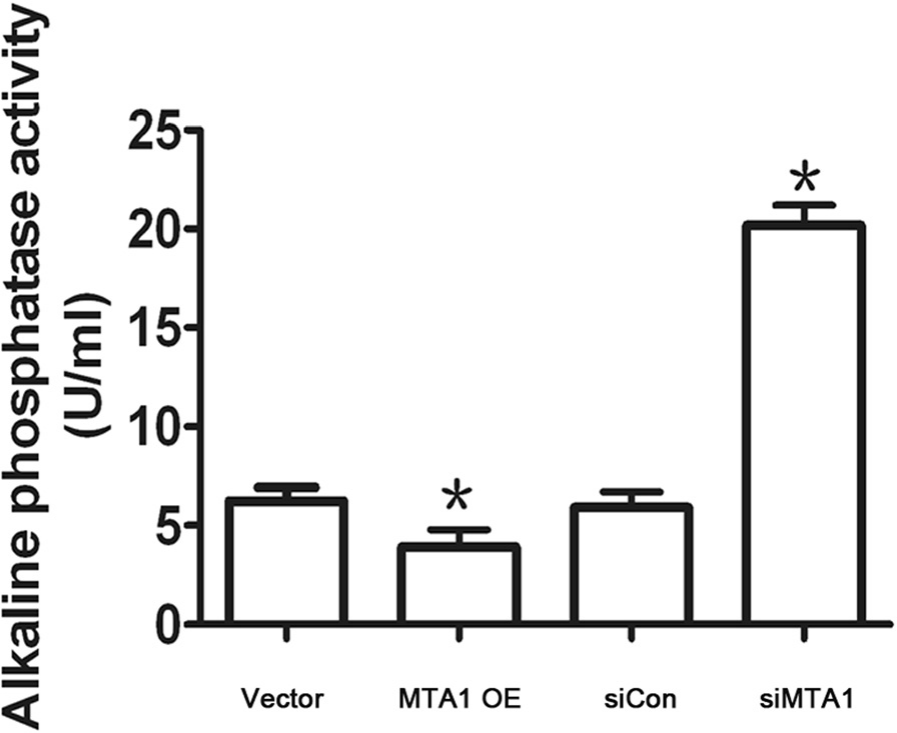
**ALP activity assay in MC3T3 cells after MTA1 knockdown or overexpression.** The ALP activity was assayed with Alkaline Phosphatase Assay Kit following the instructions. MTA1 OE, MTA1 overexpression (n = 3).

Mineral deposition is an important step for the repair of fractures, through which the structure and function of bone are rebuilt. To further clarify the role of MTA1 in osteoblast differentiation, we transfected the MC3T3 cells with MTA1-overexpressing plasmid pCDH-MTA1 or MTA1 siRNA. We found that overexpression of MTA1 significantly slowed the mineralization of osteoblast MC3T3 cells, while MTA1 silencing accelerated the process of mineralization (*P* <0.05; Figure 4). These data suggest that MTA1 is able to suppress the differentiation of osteoblast cells. ALP is a key bone cell-specific marker for osteoblast differentiation. MTA1 knockdown significantly increased ALP activity, while MTA1 overexpression decreased the activity of ALP, indicating a negative role of MTA1 in osteoblast differentiation (Figure 5).

### MTA1 alters the expression of osteocalcin, BMP-2, and ALP proteins

**Figure 6:**
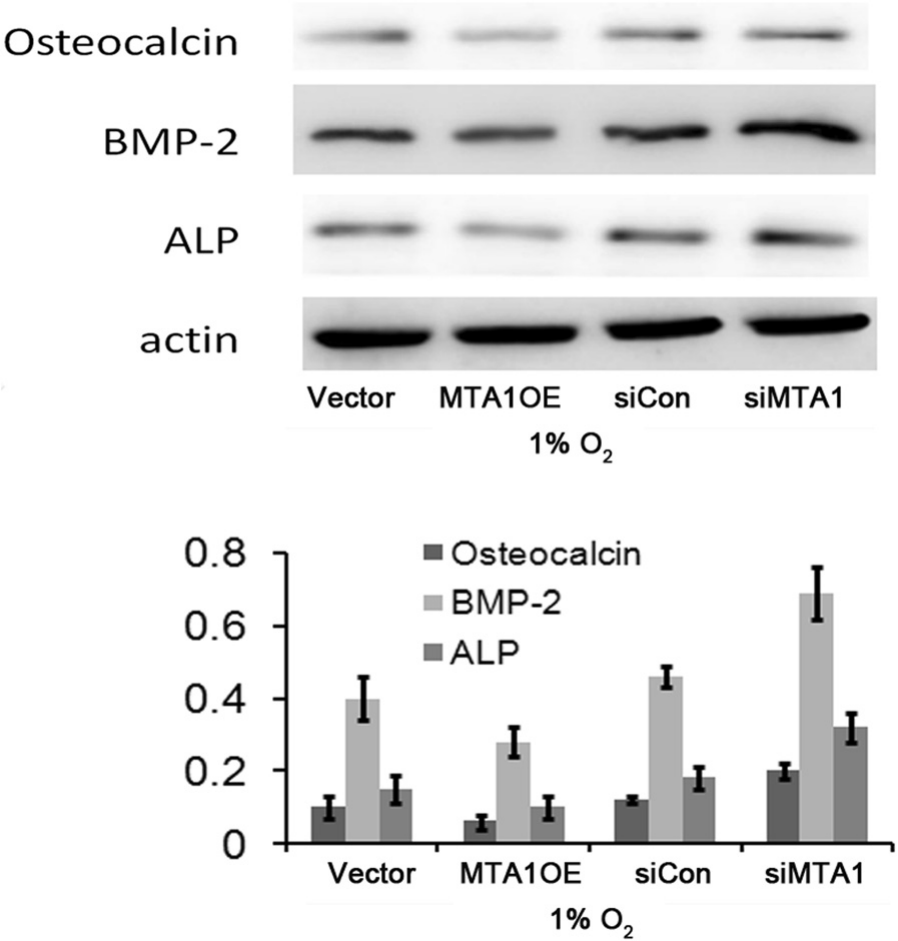
**MTA1 regulated the expression of osteocalcin, BMP-2, and ALP.** MC3T3 cells were transfected with pCDH-MTA1, MTA1 siRNA, or control siRNA. Null vector and control siRNA were used as controls. The expression of osteocalcin, BMP-2, and ALP was examined by Western blot assay. MTA1 OE, MTA1 overexpression (n = 3).

To examine whether MTA1 activation upon hypoxia altered the typical molecules involved in bone mature and repair, such as osteocalcin and BMP-2, we detected the protein levels of these molecules by Western blot. Results showed that both osteocalcin and BMP-2 were downregulated by MTA1, while they were upregulated by specific siRNA against MTA1 (Figure 6). Consistent with ALP activity assay, ALP protein level was upregulated by MTA1 siRNA but downregulated by MTA1 overexpression (Figure 6).

## Discussion

Bone fractures are one of the most frequent events occurring in all sectors of the population, from kids to youths, to adults and elders. The healing process of fractures is affected by a variety of factors, including the blood supply, oxygen concentration, and various inflammatory factors [12].

Hypoxia induced by blood supply shut-off is the main feature of a fracture, thus initiating many downstream cascades. The HIF family is one of those upstream signaling commanders. HIF-1 is a key player in hypoxia-induced physiological and pathological processes, especially in bone fractures [13]. HIF-1 is activated by hypoxia at the earliest stage of fracture and plays a positive role in the promoting the healing of bone fracture. HIF-1 is able to mediate the hypoxic-stimuli-induced expression of VEGF in MC3T3 cells [14]. The induction of VEGF in fracture conditions contributes to the re-establishment of the blood supply and hence the structure remodeling of bone.

**Figure 7:**
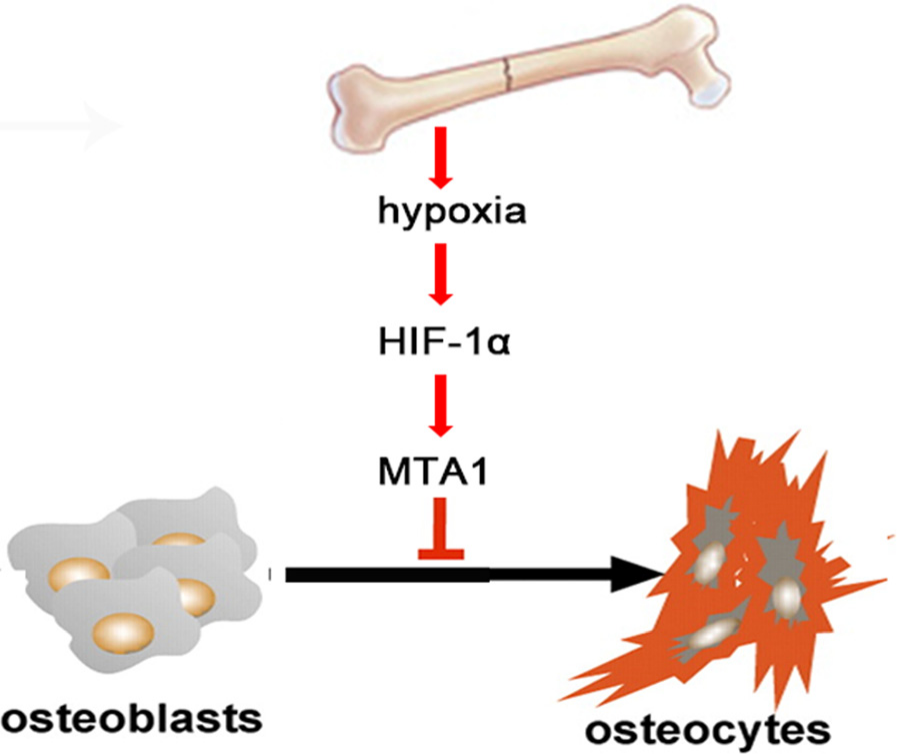
**A schematic presentation showing the signaling pathway from fracture-induced hypoxia to MTA1 upregulation, suppressing osteoblast differentiation.**

MTA1 has been mostly studied in cancer [15,16]. MTA1 protein contributes to the process of cancer progression and metastasis through multiple genes and protein targets and interacting proteins with roles in transformation, anchorage-independent growth, invasion, survival, DNA repair, angiogenesis, hormone independence, metastasis, and therapeutic resistance [17-19]. It has been reported that MTA1 regulates cancer behaviors through the Wnt pathway, which is a key knot in the stemness regulation network. Prominently, MTA1 enhances the ability of cancer cell invasion and metastasis in breast cancer and some other cancers. In estrogen receptor-positive breast cancer cells, MTA1 suppresses the estrogen-receptor element-driven transcription and disrupts estradiol responsiveness, thus contributing to progression of breast cancer to more invasive phenotypes [7]. Targeting MTA1 in the prostate blocks cancer angiogenesis [16]. As for its correlation with hypoxia conditions, it has been reported that MTA1 stabilizes HIF-1 by recruiting histone deacetylase 1, resulting in angiogenesis [20]. It is unclear whether HIF-1 has some effects on MTA1. In the current study, we found that HIF-1, as a key responder to hypoxia, is able to induce the expression of MTA1. It is the first report indicating that MTA1 may be involved in the healing process of bone fractures. We assessed the effects of HIF-1-induced MTA1 expression on biological behaviors of MC3T3 cells and found that the hypoxia-induced MTA1 upregulation had a positive effect on the healing process of bone fractures at the early stage of fracture. MTA1 promoted the growth of MC3T3 cells under hypoxia (Figure 7). The promotion of osteoblast growth by MTA1 under the hypoxic microenvironment after bone fracture is a necessary response heading to fracture healing. Moreover, assays by mineral deposition and ALP activity verified a suppressive role of MTA1 overexpression in osteoblast differentiation. The delayed differentiation of MC3T3 cells by MTA1 upregulation may promise an active proliferating osteoblast population to support the healing process. Nevertheless, the actual role of MTA1 should be verified in human osteoblasts.

Bone mineralization is regulated by a number of factors including osteocalcin, BMP-2, and ALP. We also detected the molecules involved in the fracture healing process after MTA1 manipulation. BMP-2 and osteocalcin are important molecules involved in bone repair. In MC3T3 cells, HIF-1 is able to upregulate BMP-2 [21] and downregulate osteocalcin [22]. The suppression of osteocalcin leads to a delay of osteoblast mineralization and differentiation. We found that HIF-1-mediated MTA1 expression suppressed osteocalcin expression under hypoxic conditions. ALP is a key enzyme in the process of biomineralization, inducing the mineralization of collagen sheets in the fracture. In our study, we found overexpressed MTA1 reduced ALP activity and that MTA1 knockdown resulted in increased ALP activity. Therefore, it is reasonable to propose roles of MTA1 in stimulating osteoblast growth but suppressing osteoblast differentiation at the early stage of bone fracture.

## Conclusions

It has been reported that HIF-1 is able to induce self-renewal and growth factor secretion of multipotential stromal cells under hypoxic conditions [23]. In the current study, we found that, under hypoxic conditions, the HIF-1/MTA1 pathway promoted osteoblast growth but suppressed the differentiation of MC3T3 cells, keeping the cells in a self-renewal potentiated status. This may help to accumulate more osteoblast cells at the early stage of fracture for the repair and re-construction of bone. However, the potential role of MTA1 in bone fracture healing needs to be verified in *in vivo* models.

## Additional file

Additional file 1:
**Microarray data from MC3T3 after HIF-1α silencing under hypoxia condition.**

## Abbreviations

ALP: Alkaline phosphatase
BMP-2: Bone morphogenetic protein 2
HIF: Hypoxia-inducible factor
MTA1: Metastasis tumor antigen 1
pCDH-MTA1: MTA1 coding sequence inserted into pCDH-CMV-MCS-EF1-copGFP vector
VEGF: Vascular endothelial growth factor
